# A Fluorescence-Based Competitive Antibody Binding Assay for Kynurenine, a Potential Biomarker of Kidney Transplant Failure

**DOI:** 10.3390/diagnostics12061380

**Published:** 2022-06-02

**Authors:** Max Borgolte, Isabel Quint, Lars Kaiser, René Csuk, Hans-Peter Deigner

**Affiliations:** 1 Institute of Precision Medicine, Furtwangen University, Jakob-Kienzle Str. 17, 78054 Villingen-Schwenningen, Germany; box@hs-furtwangen.de (M.B.); i.quint@hs-furtwangen.de (I.Q.); kal@hs-furtwangen.de (L.K.); 2 Institute of Organic Chemistry, Martin-Luther University Halle-Wittenberg, Kurt-Mothes-Str. 2, 06120 Halle (Saale), Germany; rene.csuk@chemie.uni-halle.de; 3 Faculty of Science, Eberhard Karls University Tuebingen, Auf der Morgenstelle 8, 72076 Tübingen, Germany; 4 Institute of Pharmaceutical Sciences, University of Freiburg, Albertstraße 25, 79104 Freiburg im Breisgau, Germany; 5 EXIM Department, Fraunhofer Institute IZI (Leipzig), Schillingallee 68, 18057 Rostock, Germany

**Keywords:** transplant failure, transplant medicine, biomarkers, fluorescent probes, fluorescence, kynurenine, amino acids, rapid testing

## Abstract

Kynurenine is a tryptophan metabolite linked to several inflammatory processes including transplant failure, a significant challenge in transplant medicine. The detection of small molecules such as kynurenine, however, is often complex and time consuming. Herein, we report the successful synthesis of a fluorescently labelled kynurenine derivative, showing proper fluorescence and anti-kynurenine antibody binding behavior in a magnetic bead immunoassay (MIA). The fluorescent kynurenine–rhodamine B conjugate shows a K_D_-value of 5.9 µM as well as IC50 values of 4.0 µM in PBS and 10.2 µM in saliva. We thus introduce a rapid test for kynurenine as a potential biomarker for kidney transplant failure.

## 1. Introduction

Kynurenine is a metabolite of the tryptophan degradation pathway linked to several inflammatory, metabolic, oncogenic [1] as well as psychiatric disorders [2,3]. While some tryptophan is metabolized to serotonin [4], 95% of the dietary tryptophan is metabolized to the kynurenine pathway (KP) via the enzyme class of indolamine-2,3-dioxygenases (IDOs) [5,6,7], followed by a cascade of degradation enzymes with quinolinic acid as the final product [8]. The activity of the IDO enzymes is regulated through immunological factors such as pathogenic microorganisms and LPS [9,10,11], inflammatory cytokines [12,13], or IL-1 and TNF-α [14]. Elevated kynurenine levels also downregulate immune activation and convey anti-inflammatory activity [15,16,17,18], demonstrating a feedback-loop-like behavior.

The link between inflammatory response and elevated kynurenine levels in serum via upregulation of IDO by pro-inflammatory factors renders kynurenine an interesting biomarker for clinically relevant inflammatory processes. Upregulated IDO and, therefore, elevated kynurenine levels of 3.9 ± 2.1 µM in serum have been found in the case of chronic kidney disease (CKD) in patients in the pre-dialysis stage. Values increase along with CKD severity up to 5.6 ± 2.3 µM because of chronic inflammation during CKD progression [19,20,21,22]. Elevated kynurenine levels are related not only to CKD but also to inflammatory processes after kidney transplantation; they are inversely correlated with kidney function [23,24]. In a study from 2007 by Buczko et al., plasma and saliva kynurenine levels from uremic patients were compared, showing reproducible results and correlations among saliva and serum kynurenine levels [25]. Due to this correlation in serum and saliva kynurenine found by Buczko et al. [25] and the association with inflammation and IDO expression both in kidney failure during chronic kidney disease and kidney transplant rejection [22,26,27], and due to the findings that kynurenine is discussed as a biomarker for renal allograft failure in the literature [28,29], here, we focus on kynurenine determined from saliva as a potential biomarker for kidney transplant rejection. In patients with transplant rejection, L-kynurenine levels of 17.4 ± 8.4 µM for serum and 4.6 ± 1.6 µM for saliva were measured, compared to control groups with serum levels of 2.7 ± 0.4 µM and 0.7 ± 0.4 µM in saliva [23,25,28,29,30].

Apart from transplant failure, elevated kynurenine levels have also been linked to titanium dental implants and to bone-osseointegration processes [31], as well as to the implantation of a left ventricular assist device, showing that kynurenine as a metabolite is linked to implant failure as well [32].

Most approaches in clinical trials to quantifying kynurenine from different tissues are focused on LC-MS [33,34,35] or GC-MS [36] methods, displaying clinical impracticability and delayed diagnosis. Apart from these, Ungor et al., 2019, presented fluorescent gold nanoclusters able to detect kynurenine in physiological concentrations in PBS with a quenching mechanism [37]. Another approach is the usage of fluorescently labelled kynurenine derivatives, as shown by Klockow et al., 2013, who labelled kynurenine with a coumarin aldehyde scaffold, showing fluorescence while undergoing a shift in pH [38]. Other approaches for potential rapid testing, using the kynurenine pathway for diagnosis, focus on quantifying the IDO activity instead of measuring kynurenine directly [39].

To date and due to the small size of the kynurenine molecule, no fluorescently labelled kynurenine derivatives bound to an anti-kynurenine antibody are described in the literature. Likewise, there is no functional bioassay based on antibody binding of kynurenine described in the literature. Herein, we report the synthesis of a fluorescent, rhodamine B-labelled kynurenine derivative and demonstrate its binding ability to an anti-kynurenine antibody. We demonstrate that this denotes a promising approach for the development of a competitive kynurenine antibody-binding assay and ultimately a kynurenine rapid test from saliva and possibly other body fluids.

## 2. Materials and Methods

### 2.1. General Experimental

TLC was carried out on Silica Gel 60 F254 (Merck, layer thickness 0.2 mm) with detection by UV light (254 nm) or by charring with 1% KMnO_4_ in 1N NaOH. Flash column chromatography (FC) was performed on M&N Silica Gel 60 (0.063–0.200 mm). ^1^H NMR and ^13^C NMR spectra were recorded on a Bruker Avance I 200 (200 MHz), Bruker Avance II 400 (400 MHz, both Bruker, Billerica, MA, USA), or Varian Unity 500 (500 MHz) spectrometer (Varian Inc., Palo Alto, CA, USA). Chemical shifts are reported in parts per million relative to solvent signals (CDCl_3_: δH = 7.26 ppm, δC = 77.0 ppm; DMSO-d6: δH = 2.49 ppm, δC = 39.7 ppm). Signals were assigned by first-order analysis and assignments were supported where feasible by 2-dimensional ^1^H, ^1^H and ^1^H, ^13^C correlation spectroscopy. Coupling constants are reported in hertz. UV/vis spectra were recorded on a PerkinElmer Lambda XLS+ UV/vis spectrometer (PerkinElmer, Waltham, MA, USA) in a 10.00 mm QS quartz cuvette. Fluorescence was recorded on a Tecan Infinite M200 multiplate reader in commercially available 96-well multitier plates. Chemicals and reagents were purchased from Acros Organics, Alfa Aesar, Sigma-Aldrich, Carl Roth, Carbolution or ABCR and were used without further purification.

### 2.2. Synthesis of Kynurenine–Rhodamine B Conjugates

#### 2.2.1. [6-Diethylamino-9-(2-prop-2-ynyloxycarbonyl-phenyl)-xanthen-3-ylidene]-diethyl-ammonium; Chloride **1**

Rhodamine B (10 g, 20.9 mmol, 1 eq) was dissolved in dry CH_2_Cl_2_ (250 mL) under N_2_ atmosphere. EDC (4.4 g, 22.99 mmol, 1.1 eq) and DMAP (0.51 g, 4.18 mmol, 0.2 eq) were added, and the mixture obtained was stirred for 15 min at room temperature. After the addition of propargyl alcohol (1.33 mL, 22.99 mmol, 1.1 eq) and after stirring overnight at room temperature, the mixture was washed 2x with 1M HCl (250 mL) and 1x with brine (250 mL). Drying the organic layer over Na_2_SO_4_ and evaporating the solvent yielded the crude product. Purification via flash column chromatography (CH_2_Cl_2_: MeOH 9:1) gave the final product: violet powder (6.08 g, 56%).

^1^H-NMR (CDCl_3_, 400 MHz): 8.66 (d, *J* = 7.9 Hz, 1 H, Ar-H), 7.88 (t, *J* = 7.6 Hz, 1 H, Ar-H), 7.78 (t, *J* = 7.7 Hz, 1 H, Ar-H), 7.38 (d, *J* = 7.6 Hz, 1 H, Ar-H), 7.09 (d, *J* = 9.4 Hz, Ar-H, 1 H), 6.95 (d, *J* = 9.6 Hz, Ar-H, 1 H), 6.88 (s, Ar-H, 1 H), 4.65 (d, *J* = 1.3 Hz, CH_2_, 2 H), 3.68 (q, *J* = 7.1, CH_2_, 8 H), 2.46 (s, CH, 1 H), 1.36 (t, *J* = 7.1, CH_3_, 12 H).

^13^C-NMR (DMSO, 50 MHz): 164.4 (C(=O)O), 157.6 (Ar-C), 155.6 (Ar-C), 134.0 (Ar-C), 133.8 (Ar-C), 131.3 (Ar-C), 131.3 (Ar-C), 131.0 (Ar-C), 129.3 (Ar-C), 115.1 (Ar-C), 113.4 (Ar-C), 107.4 (Ar-C), 96.4 (Ar-C), 78.5 (C), 77.8 (CH), 53.3 (CH_2_), 45.8 (CH_2_), 12.9 (CH_3_).

#### 2.2.2. 4-(2-Amino-phenyl)-2-tert-butoxycarbonylamino-4-oxo-butyric Acid **2**

NH_2_ protection of kynurenine was carried out by Boc-chemistry following Schotten–Baumann conditions: kynurenine (500 mg, 2.4 mmol, 1 eq) was dissolved in a water/THF 1:1 mixture containing NaOH (288 mg, 7.2 mmol, 3 eq). After the solution turned clear, Boc_2_O (1.55 mL, 7.2 mmol, 3 eq) was added dropwise at 0 °C, and the reaction was monitored by TLC. Complete conversion was observed after 90 min. Acidification using 10% HCl, followed by 3 extractions with ethyl acetate, drying of the organic layer and evaporation of the solvent yielded the crude product: a yellowish oil. Column chromatography (ethyl acetate) yielded the pure product: a yellowish powder (327.7 mg, 44%).

^1^H-NMR (400 MHz, CDCl_3_): 7.73 (d, *J* = 8.0 Hz, 1H- Ar-H), 7.34 (t, *J* = 7.6 Hz, 1H, Ar-H), 6.83–6.72 (m, 2H, Ar-H), 5.64 (d, *J* = 8.7 Hz, 1H, CH), 4.67 (ddd, *J* = 12.8, 8.5, 4.3 Hz, 1H, CH), 3.80–3.69 (m, 2H, CH2), 3.53 (dd, *J* = 18.0, 3.8 Hz, 1H, CH), 1.45 (s, 9H, 3x CH3).

^13^C-NMR (100 MHz, CDCl_3_): 199.54 (Ar-C(=O)), 172.43 (C(C=O)OH), 155.68 (Boc-C(=O)), 149.45 (Ar-C), 134.96 (Ar-C), 131.08 (Ar-C), 125.83 (Ar-C), 118.10 (Ar-C), 116.73 (Ar-C), 69.98 (Boc-*tert*-C), 52.60 (CH), 41.47 (CH_2_), 28.34 (CH_3_).

#### 2.2.3. [3-(2-Amino-phenyl)-1-(2-{2-[2-(2-azido-ethoxy)-ethoxy]-ethoxy}-ethylcarbamo-yl)-3-oxo-propyl]-carbamic Acid Tert-Butyl Ester **3**

Boc-protected kynurenine **1** (318.9 mg, 1.03 mmol, 1 eq) and the corresponding azido-linker (248.68 mg, 1.14 mmol, 1.1 eq) were dissolved in CH_2_Cl_2_ (50 mL). EDC (197.45 mg, 1.03 mmol, 1 eq) and DMAP (25.17 mg, 0.206 mmol, 0.2 eq) were added, and the mixture was stirred at room temperature for 48 h. Washing 3× with 2 M NaOH (80 mL) followed by washing with brine (80 mL), drying over Na_2_SO_4_ and evaporation yielded the crude product. Column chromatography (ethyl acetate) yielded the pure product: an orange solid, which was used directly in the next step without further analysis (175.11 mg, 33%).

#### 2.2.4. (9-{2-[1-(2-{2-[4-(2-Amino-phenyl)-2-tert-butoxycarbonylamino-4-oxo-butyrylamino]-ethoxy}-ethyl)-1H-[1,2,3]triazol-4-ylmethoxycarbonyl]-phenyl}-6-diethylamino-xanthen-3-ylidene)-diethyl-ammonium Salt **4**

Azido functionalized Boc-L-Kynurenine **3** (166 mg, 326.6 µmol, 1 eq) and propargyl rhodamine B **1** (169 mg, 326.6 µmol, 1 eq) were dissolved in 30 mL of a mixture of CH_2_Cl_2/_MeOH/H_2_O 10:10:3. After adding an aqueous CuSO_4_ solution (262 µL, 0.5 M, 130.64 µmol, 0.4 eq), TBTA (18 mg, 32.66 µmol, 0.1 eq) and Na ascorbate (142 mg, 718.52 µmol, 2.2 eq), the mixture was heated to 60 °C for 16 h. After cooling down, 20 mL of ddH_2_O was added and the mixture was extracted 3 times with 50 mL CH_2_Cl_2_, followed by drying over Na_2_SO_4_ and evaporation of the solvent. The pure product was obtained after column chromatography (CH_2_Cl_2_/MeOH 3:1) as a pink powder (302.5 mg, 90%).

^1^H-NMR (DMSO, 500 MHz): 8.20 (dd, *J* = 7.9, 1.0 Hz, 1H, Ar-H), 7.91–7.85 (m, 3H, Ar-H), 7.83–7.79 (m, 2H, Ar-H), 7.47 (dd, *J* = 7.6, 0.8 Hz, 1H, Ar-H), 7.08–7.00 (m, 2H, Ar-H), 6.97–6.90 (m, 5H, Ar-H), 5.04 (s, 2H, Ar-CH_2_), 4.65 (d, *J* = 2.4 Hz, 1H, CH_2_), 4.44 (t, *J* = 5.2 Hz, 2H, Triazol-CH_2_), 3.74 (t, *J* = 5.2 Hz, 2H, O-CH_2_), 3.63 (dd, *J* = 13.9, 6.7 Hz, 8H, 4x RhB-CH_2_), 3.48–3.45 (m, 2H, O-CH_2_), 3.44–3.40 (m, 6H, 3× O-CH_2_), 3.30 (t, *J* = 6.4 Hz, 2H, O-CH_2_), 3.00 (dd, *J* = 12.0, 6.0 Hz, 2H, CH_2_), 1.33 (s, 9H, 3× Boc-CH_3_), 1.20 (t, *J* = 6.7 Hz, 12H, 4x RhB-CH_3_).

^13^C-NMR (DMSO, 125 MHz): 198.9 (Ar-C(=O), 172.1 (C(=O)-NH), 164.9 (C(=O)-O), 157.8 (Ar-H), 157.5 (Boc-C(=O)), 156.0 (Ar-C), 155.6 (Ar-C), 155.5 (Ar-C), 141.04 (Triazol-Ar-C), 134.6 (Ar-C), 133.9 (Ar-C), 133.8 (Ar-C), 133.7 (Ar-C), 131.3 (Ar-C), 131.2 (Ar-C), 131.0 (Ar-C), 130.9 (Ar-C), 129.6 (Ar-C), 129.2 (Ar-C), 125.3 (Triazol-Ar-C), 115.0 (Ar-C), 114.9 (Ar-C), 114.8 (Ar-C), 113.3 (Ar-C), 96.3 (Ar-C), 78.4 (*tert.*-C), 78.0 (NH(Boc)-CH), 77.8 (O-CH_2_), 70.1 (O-CH_2_), 70.0 (O-CH_2_), 69.9 (O-CH_2_), 69.6 (O-CH_2_), 69.3 (O-CH_2_), 68.0 (O-CH_2_), 58.6 (Ar-CH_2_), 53.2 (RhB-CH_2_), 49.8 (Ar-CH_2_), 45.8 (N-CH_2_), 28.7 (Boc-CH_3_), 12.9 (RhB-CH_3_).

#### 2.2.5. (9-{2-[1-(2-{2-[2-Amino-4-(2-amino-phenyl)-4-oxo-butyrylamino]-ethoxy}-ethyl)-1H-[1,2,3]triazol-4-ylmethoxycarbonyl]-phenyl}-6-diethylamino-xanthen-3-ylidene)-diethyl-ammonium Salt **5**

Boc-deprotection of rhodamine B–kynurenin conjugate **4** was carried out by dissolving conjugate **4** in 6 mL of CH_2_Cl_2_ containing 25% trifluoroacetic acid. After stirring at room temperature for 1 h, the solution was precipitated in 50 mL ice cold Et_2_O and centrifuged for 5 min at 4 °C and max speed. The precipitate was dissolved in ddH_2_O and lyophilized. Purification by HPLC yielded the pure product: a pink solid (60.5 mg, 26%).

^1^H-NMR (DMSO, 500 MHz): 8.20 (dd, *J* = 8.0, 1.4, 1H, Ar-H), 8.11 (s, 1H, C(=O)NH), 7.93–7.84 (m, 3H, Ar-H), 7.85–7.78 (m, 2H, Ar-H), 7.47 (dd, *J* = 7.7, 1.4, 1H, Ar-H), 7.05–6.99 (m, 3H, Ar-H), 6.97–6.89 (m, 5H, Ar-H), 5.05 (d, *J* = 4.4, 3H, Ar-CH_2_ + CH), 4.47–4.41 (m, 2H, Ar-CH_2_), 3.75 (t, *J* = 5.2, 2H, O-CH_2_), 3.63 (q, *J* = 6.9, 8H, RhB-CH_2_), 3.54 (d, *J* = 5.6, 2H, O-CH_2_), 3.50–3.42 (m, 8H, O-CH_2_), 2.92 (q, *J* = 5.6, 2H, O-CH_2_), 1.20 (t, *J* = 7.0, 12H, RhB-CH_3_).

^13^C-NMR (DMSO, 125 MHz): 197.3 (Ar-C(=O)), 168.8 (C(=O)-NH), 164.9 (C(=O)-O), 158.2 (Ar-C), 157.9 (Ar-C), 157.5 (Ar-C), 155.5 (Ar-C), 141.1 (Triazol-Ar-C), 133.2 (Ar-C), 133.7 (Ar-C), 131.2 (Ar-C), 130.9 (Ar-C), 129.6 (Ar-C), 125.3 (Triazol-Ar-C), 117.5 (Ar-C), 114.9 (Ar-C), 113.3 (Ar-C), 96.3 (Ar-C), 70.1 (O-CH_2_), 70.0 (O-CH_2_), 69.9 (O-CH_2_), 69.0 (O-CH_2_), 67.1 (O-CH_2_), 58.6 (Ar-CH_2_), 49.8 (Ar-CH_2_), 45.7 N-CH_2_), 12.87 (CH_3_).

#### 2.2.6. (6-Diethylamino-9-{2-[1-(2-{2-[2-(2-hydroxy-ethoxy)-ethoxy]-ethoxy}-ethyl)-1H-[1,2,3]tr-iazol-4-ylmethoxycarbonyl]-phenyl}-xanthen-3-ylidene)-diethyl-ammonium Salt **6**

Propargyl rhodamine B **1** (100 mg, 193.4 µmol, 1 eq) and azidotetraethylene glycol (42.4 mg, 193.4 µmol, 1 eq, synthesized according to the literature [40]) were dissolved in 25 mL of a mixture of CH_2_Cl_2/_MeOH/H_2_O 10:10:3. A solution of CuSO_4_ in H_2_O (0.5 M, 15.5 µL, 7.74 µmol, 0.04 eq) was added, TBTA (1 mg, 1.93 µmol, 0.01 eq) and Na ascorbate (8.4 mg, 42.6 µmol, 0.22 eq) were added, and the mixture was heated to 60 °C. The reaction was monitored by TLC. After 16 h, the mixture was left to cool down, followed by the addition of 25 mL of ddH_2_O. Extraction with 50 mL of CH_2_Cl_2_ 3 times, followed by drying over Na_2_SO_4_, gave the crude product. Column chromatography (CH_2_Cl_2_/MeOH 3:1) gave the pure product: a pink oil (135.8 mg, 95%).

^1^H-NMR (DMSO, 500 MHz): 8.22 (d, *J* = 8.0 Hz, 1H, Ar-H), 7.89 (t, *J* = 5.2 Hz, 2H, Ar-H), 7.82 (t, *J* = 7.9 Hz, 1H, Ar-H), 7.48 (d, *J* = 7.5 Hz, 1H, Ar-H), 7.04 (dd, *J* = 9.5, 2.2 Hz, 2H, Ar-H), 6.96 (s, 1H, Ar-H), 6.95–6.92 (m, 3H, Ar-H), 5.06 (s, 2H, Ar-CH_2_), 4.56 (s, 1H, OH), 4.45 (t, *J* = 5.2 Hz, 2H, O-CH_2_), 3.76 (t, *J* = 5.2 Hz, 2H, O-CH_2_), 3.65 (q, *J* = 6.8 Hz, 8H, CH_2_), 3.49 (dd, *J* = 5.6, 3.1 Hz, 2H, O-CH_2_), 3.46–3.41 (m, 6H, O-CH_2_), 3.37–3.34 (m, 2H, O-CH_2_), 1.22 (t, *J* = 6.8 Hz, 12H, CH_3_).

^13^C-NMR (DMSO, 125 MHz): 164.88 (C(=O)), 157.88 (Ar-H), 157.53 (Ar-H), 155.53 (Ar-H), 141.05 (Ar-H), 133.71 (Ar-H), 131.24 (Ar-H), 130.93 (Ar-H), 129.67 (Ar-H), 125.36 (Ar-H), 114.94 (Ar-H), 113.32 (Ar-H), 96.29 (Ar-H), 72.75 (CH_2_), 70.21 (CH_2_), 70.17 (CH_2_), 70.05 (CH_2_), 69.97 (CH_2_), 69.01 (CH_2_), 66.81 (CH_2_), 60.63 (CH_2_), 58.63 (CH_2_), 49.77 (CH_2_), 45.76 (CH_2_), 12.89 (CH_3_).

### 2.3. Magnetic Bead Immunoassay (MIA)

#### 2.3.1. Antibody Biotinylation

A total of 1 µL of a 6 mg/mL Biotin-NHS solution (NHS-dPEG^®^12-biotin, Sigma Aldrich, Taufkirchen, Germany) was added to 100 µL of an anti-kynurenine antibody (monoclonal Mouse IgG1a k chain anti-kynurenine antibody, clone 3D4-F2, ImmuSmol SAS, 0.5 mg/mL). The mixture was incubated for 50 min at room temperature with gentle shaking. Excess Biotin-NHS was removed using a VivaSpin 500 centrifugal concentrator with 10 kDa MWCO (Sigma Aldrich).

#### 2.3.2. Bead Preparation

For bead activation, 50 µL of magnetic beads (Dynabeads™ MyOne™ Streptavidin C1 magnetic beads 10 mg/mL, Thermo Fisher Scientific, Schwerte, Germany) was diluted to 1 mg/mL with 450 µL of PBS and pelleted on a magnetic rack for 2 min. The supernatant was discarded, and the beads were washed three times with 500 µL PBS. After the last washing step, 20 µL of the supernatant was replaced with 20 µL biotinylated antibody (0.5 mg/mL). The mixture was incubated for 30 min at RT under gentle shaking. The reaction was blocked by washing the beads three times with PBS containing 1.5% BSA and 0.5% Tween-20. The final concentration was 20 µg of antibody per 1 mg of beads.

#### 2.3.3. Immunoassay Conjugate Binding

For 3 h at RT, 0–100 µM rhodamine B–kynurenine conjugate **5** or rhodamine B-PEG-Linker **6** was incubated with 50 µL of antibody-conjugated magnetic beads and 50 µL of PBS (1:3 dilution). The beads were pelleted on a magnetic rack, and the unbound rhodamine B in the supernatant was quantified in a 96-well-plate with a fluorescence measurement of 100 µL of supernatant at 561 nm excitation and 592 nm emission, using a Tecan Infinite M200 multiplate reader. A standard curve of rhodamine B fluorescence intensity between 0 and 100 µM diluted 1:3 in PBS was used for the calculation of bead-bound conjugate **5** or **6**.

#### 2.3.4. Competition between Rhodamine B–Kynurenine Conjugate and Native Kynurenine

Fifty microliters of antibody coupled beads was incubated with 50 µL of 12 µM rhodamine B-kynurenine conjugate **5** and 50 µL of spiked PBS or artificial saliva (Sigma Aldrich, SAE0149) containing 0–250 µM native L-kynurenine for 3 h at RT on a hula shaker (1:3 dilution). Beads were pelleted on a magnet and fluorescence intensity of unbound rhodamine B–kynurenine conjugate 5 in 100 µL supernatant was measured in a Tecan Infinite M200 multiplate reader at 561 nm excitation and 592 nm emission. Bead-bound L-kynurenine was indirectly calculated by calculating the amount of displaced rhodamine B–kynurenine conjugate **5**. Therefore, the fluorescence intensity of bound rhodamine B–kynurenine conjugate **5** without L-kynurenine was subtracted from the fluorescence intensity of samples with different L-kynurenine concentrations.

## 3. Results and Discussion

### 3.1. Synthesis of Fluorescent Kynurenine Conjugates

**Figure 1:**
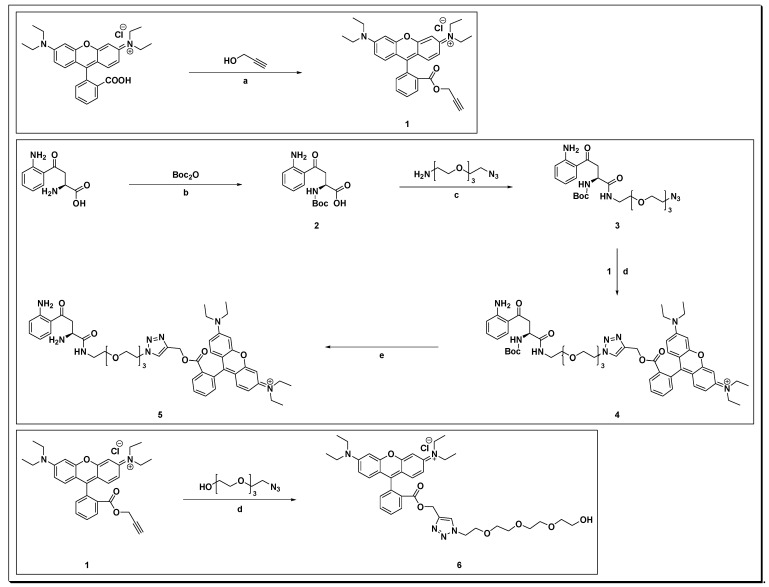
Route of synthesis for the fluorescent labelled kynurenine probe. Rhodamine B was reacted with propargyl alcohol to rhodamine B propargyl ester **1**, which was reacted with an azido tetraethylene glycol linker to afford **6** or with azido-kynurenine to yield product **5**. Reaction conditions: (a) EDC, DMAP, CH_2_Cl_2_, RT, overnight; (b) NaOH, H_2_O/THF 1:1, RT, 90 min; (c) EDC, DMAP, CH_2_Cl_2_, RT, 48 h; (d) 1, CuSO_4_, TBTA, Na ascorbate, H_2_O/MeOH/CH_2_Cl_2_ 10:10:3, 16 h; and (e) CH_2_Cl_2_/TFA 4:1, RT, 1 h.

Chemical synthesis of the fluorescent L-kynurenine–rhodamine B conjugate was carried out as shown in Figure 1. First, commercial rhodamine B was reacted with propargyl alcohol to the corresponding rhodamine B propargyl ester **1** using Steglich esterification by EDC and DMAP, which was used as the fluorescent probe for click-conjugates **5** and **6**.

For the azido tetraethylene glycol L-kynurenine derivative **3**, Boc-protection of the primary amine of kynurenine was carried out to obtain product **2**, followed by attaching an amino-azido-tetraethylene glycol linker, synthesized according to a published protocol [41] yielding the azido-4EG-L-kynurenine derivative **3**. Click reaction of **3** with **1** gave product **4**, followed by Boc-deprotection to the fluorescent kynurenine probe **5**. To study the effect of the linker as well as the rhodamine B moiety on antibody binding, rhodamine B clickamer **6** containing only a tetraethylene glycol linker [40] was prepared, following the same reaction conditions as for product **4**.

### 3.2. Spectral Properties

**Figure 2:**
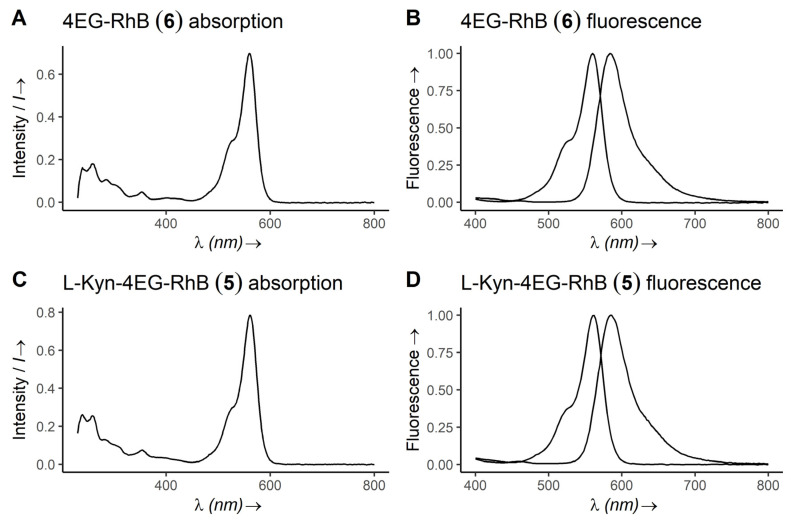
Absorption (**A**,**C**) and fluorescence (**B**,**D**) spectra of compounds **5** and **6**, measured in ddH_2_O. Absorption maxima of compounds **5** and **6** are shifted slightly to 560 nm in the comparison at 554 nm for native rhodamine B [42]. Fluorescence maxima are 586 nm.

To determine emission and absorption maxima for the synthesized rhodamine B click-conjugates, UV/vis spectra of the compounds were recorded in ddH_2_O (cf. Figure 2). The absorption maximum of rhodamine B is at 554 nm [42] while the absorption maxima of the rhodamine B conjugates **5** and **6**, containing a benzoic ester instead of a free benzoic acid at the rhodamine’s benzoic acid residue, is slightly shifted to 560 nm. Emission maxima were determined to be at 586 nm for the L-Kyn-4EG-RhB probe **5**, while the 4EG-RhB clickamer **6** shows an emission maximum of 584 nm, indicating suitable absorption and emission properties for immunoassays.

### 3.3. Magnetic Bead Immunoassay (MIA)

**Figure 3:**
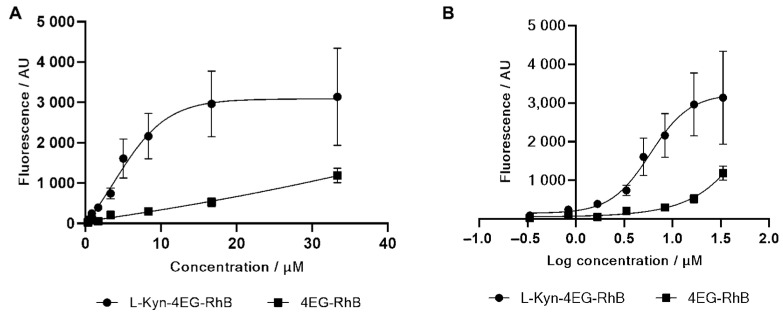
Binding assay of L-Kyn-4EG-RhB conjugate **5** and 4EG-RhB control **6** with antibody-coated magnetic beads (**A**) Concentration in micromole per liter and (**B**) logarithmic analyte concentration. Binding of the conjugate **5** to the antibody is significantly stronger than binding of the 4EG-RhB control **6**. A specific binding of the conjugate to the antibody is concluded.

To determine the antibody binding capabilities of the synthesized fluorescent L-kynurenine conjugate, we elucidated the antibody binding capability of fluorescent conjugates **5** and **6** as well as their competitive binding capability by using magnetic bead-bound anti-kynurenine antibodies, followed by incubation with the fluorescent conjugates and the subsequent fluorescence measurements of unbound conjugate in the supernatant. The tetraethylene glycol rhodamine B clickamer **6** was used as a negative control to exclude unspecific interactions between the rhodamine B or the tetraethylene glycol linker with the antibody. Antibody binding data are shown in Figure 3. As the magnetic beads are suspended in solution, the surface coated with antibodies is effectively increased, leading to increased assay sensitivity and favorable binding kinetics and, therefore, more accurate data on competition between fluorescent probe **5** and native L-kynurenine.

**Figure 4:**
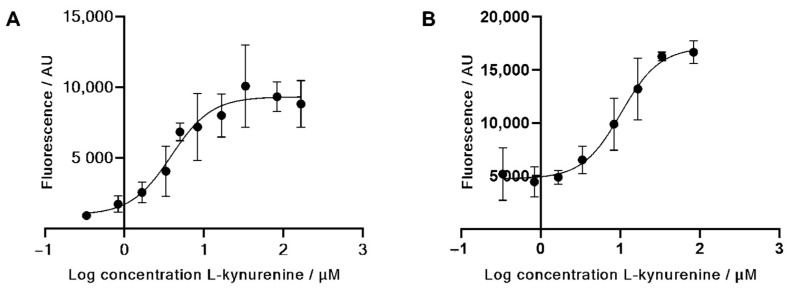
Competitive antibody binding assay in magnetic bead assay of the L-kynurenine–rhodamine B conjugate **5** against native L-kynurenine in (**A**) PBS and (**B**) artificial saliva. Fluorescence of the solution increases with increasing L-kynurenine concentration, meaning native kynurenine competes in antibody binding against the kynurenine conjugate.

To this end, antibodies were bound to magnetic beads in 1 µm diameter, followed by incubation with the target compounds, pelleted via magnet and the fluorescence measured in the supernatant. In the competitive assay, 0–83 µM L-kynurenine was incubated together with either 4 µM RhB-4EG-L-Kyn conjugate **5**, followed by a pelleting of the beads and the measurement of the fluorescence in the supernatant. The binding curves of **5** and **6** as well as the competitive binding curve are shown in Figure 3 and Figure 4.

With increasing concentration of the fluorescent L-kynurenine conjugate **5**, the measured fluorescence in the supernatant decreases since more fluorescent conjugates bind to the antibody. Figure 3A shows the difference in fluorescence intensity between L-kynurenine conjugate **5** and 4EG-RhB clickamer **6** with and without incubation with the bead-bound antibodies. In higher concentrations, the 4EG-RhB clickamer **6** binding affinity also increases, probably due to unspecific interactions between either the linker or the rhodamine B residue. Apart from this, the difference in measured fluorescence for the L-kynurenine conjugate **5** is significantly stronger, showing a K_D_-value of 5.9 µM, and therefore, unspecific interactions between the 4EG-RhB clickamer **6** and the magnetic bead-bound antibody can be neglected.

As shown in Figure 4, the competitive binding assay shows a proper increase in fluorescence along with increasing concentrations of native L-kynurenine in both PBS and artificial saliva. Increasing fluorescence levels are caused by the displacement of the fluorescent conjugate through native kynurenine. The IC_50_ values of this competition were calculated to be 4.0 µM in PBS and 10.2 µM in saliva. As expected, the IC_50_ value in saliva is higher than in PBS due to interfering components such as enzymes and proteins in saliva samples.

## 4. Conclusions

We successfully synthesized a fluorescent kynurenine conjugate based on rhodamine B with unique antibody binding properties, a promising component of a future rapid diagnostic test for kynurenine, a metabolite with a clinically relevant marker for the diagnosis of distinct diseases. Spectral properties of the products were elucidated, showing only a slight shift of 6 nm in the fluorescence emission maximum when compared to native rhodamine B. The antibody binding was investigated, and the magnetic bead assay showed a good sensitivity with a K_D_-value of 5.9 µM for the L-kynurenine conjugate **5** and IC50 values of 4.0 µM in PBS and 10.2 µM in saliva for the competitive assay. Since an increase in kynurenine levels in saliva to 4.6 ± 1.6 µM under pathological conditions, compared to 0.7 ± 0.4 µM in the healthy subject, are observed [30], it thus is possible to detect metabolite changes with statistical significance by using repeated measurements. This allows for the detection of transplant rejection in a clinical setting subject to validation in clinical trials. In addition, this approach offers the possibility of using other body fluids such as blood serum, where kynurenine levels are much higher with 17.4 ± 8.4 µM for serum compared to 4.6 ± 1.6 µM for saliva [30], even though the influence of matrix proteins and other metabolites from serum would have to be investigated in more detail for this to obtain reliable measurements. Since the current standard methods for kynurenine detection are based on liquid chromatography, they are not practical for routine diagnostics; likewise, no clinically applied functional bioassay based on antibody binding of kynurenine exists. Together, our investigations provide a promising approach towards future rapid tests for kynurenine. Here, we introduced a microbead competitive assay allowing for the determination of L-kynurenine metabolites directly from saliva, thus avoiding the use of invasive procedures and expensive equipment.

## Data Availability

Not applicable.
